# "I'm pregnant and I have breast cancer"

**DOI:** 10.1186/1471-2407-7-93

**Published:** 2007-05-30

**Authors:** Michael J Naughton, Matthew Ellis

**Affiliations:** 1 Siteman Cancer Center, Washington University School of Medicine, St. Louis, MO 63110, USA

## Abstract

Pregnancy is infrequently complicated by the diagnosis of a concurrent breast cancer. This presents a particularly complicated clinical problem. The treatment of breast cancer in young women involves a number of difficult decisions regarding therapy. These decisions become even more complex when the concerns of the safety of an unborn child are added to the equation. For breast cancers diagnosed late in the third trimester, it is relatively straight forward to delay therapy until after delivery. For women diagnosed earlier in pregnancy, there are legitimate concerns that delays in therapy may adversely affect outcomes. While there are no randomized trials addressing the optimal treatment of women in this situation, there are case reports, case series, and cohort experiences that provide some insight. There are recommendations available from an international working group and from the National Comprehensive Cancer Network that address the treatment of women in this situation. There is general consensus that both surgery and chemotherapy are relatively safe after the first trimester of pregnancy. It is generally agreed that therapeutic radiation, if necessary, should be delayed until completion of pregnancy.

"I'm pregnant" is a phrase most women share with their partners and family with great joy, anticipation, and exuberance. "I have breast cancer" is a phrase all women dread. A small number of women experience both sets of emotions in quick succession after being diagnosed with breast cancer in early pregnancy. It is currently estimated that 1 in 3000 pregnancies is complicated by breast cancer [1] and this number is expected to rise with the tendency of women to delay child bearing in developed countries [2,3].

In an article published in *BMC Cancer*, Dr. Epstein addresses many of the uncertainties that confront a patient and care team faced with this situation [4]. Here, we attempt to provide some guidance regarding currently accepted approaches to this complex situation.

The diagnosis of breast cancer during pregnancy presents a unique clinical challenge. The management of breast cancer involves a number of important decisions of high complexity, often requiring the collaboration of patient, family, and interdisciplinary care team. Add an early pregnancy into the equation and the complexity increases dramatically. This is in no small part due to the probabilistic nature of many of the decisions made in the treatment of breast cancer. The data we use to make decisions in the treatment of breast cancer is incomplete and evolving. In the setting of pregnancy, this is even more difficult, as the existing data is even less complete, and the additional issues concerning the safety of the unborn child need to be considered.

The goals of treating breast cancer in pregnancy are the same as in any other setting, preserving the life and health of the woman affected with breast cancer. In woman with local or regional disease, this means local and systemic control of her disease. If the pregnancy is to be continued, the treatment of the breast cancer may need to be modified minimize potential fetal toxicity. There is some consensus that both surgery and chemotherapy can be utilized during pregnancy, with some caveats [5]. The role of radiation during pregnancy is more controversial [6], but if required therapeutic radiation can often be delayed until after delivery without disrupting the usual treatment algorithm for the treatment of breast cancer. Hormone therapy, if indicated, must wait until the completion of the pregnancy.

A woman diagnosed with breast cancer in early pregnancy has three basic options; terminate the pregnancy, delay all treatment until after completion of pregnancy, continue the pregnancy and proceed with a modified treatment plan for her breast cancer based upon best available evidence. While terminating the pregnancy is an option, there is not data that this improves the probability of a good cancer outcome [7].

Delaying treatment until after delivery is a reasonable option for women diagnosed late in the third trimester. However for women diagnosed earlier in pregnancy, a prolonged delay in therapy potentially diminishes the probability of cancer survival. Women diagnosed with breast cancer during pregnancy tend to have high grade cancer, with many having lymphovascular space invasion [8]. The testing of hormone receptors during pregnancy is controversial, but similar to age matched controls, many pregnant women are reported to be receptor negative [9]. In addition, women diagnosed with breast cancer during pregnancy tend to have more advanced disease compared to non-pregnant women [10]. Biologic factors leading to more rapid tumor growth may account for this observation [9,10]. These characteristics make the decision to delay therapy worrisome, as the probability of upstaging occurring during a delay may be higher than in non-pregnant patients.

Proceeding with a modified treatment plan during pregnancy represents the third and most frequently applied option. Breast cancer surgery during pregnancy is considered relatively safe based upon available case series [11,12]. Surgery is often delayed until after the first trimester due to the high rate of spontaneous abortion in the first trimester. The experience with chemotherapy during pregnancy is certainly limited. Based upon the available literature, which is restricted to case reports, case series, and one cohort study [13,14] the safety of chemotherapy for mother and fetus is reasonable in the second and third trimester. For instance, the rate of major congenital malformations for chemotherapy during pregnancy has been reported to be 3%, while that for the general population is reported as 2–3% [15]. The regimens for which the most information is available are the anthracycline containing regimens, such as FAC and AC. While contemporary chemotherapy might include a taxane, anthracycline containing regimens are of proven efficacy, and based upon the treatment schedule, a taxane could likely be given in sequence after delivery.

Treatment recommendations are available based upon a meeting of international experts as well as from the National Comprehensive Cancer Network^®^; giving some guidelines based upon the best available evidence [5,16]. It is incumbent upon us to continue to improve the limited knowledge we have regarding this complex issue. Randomized trials will not be able to address this situation, and continued observation and reassessment of outcomes of the women who encounter this situation through national or international registry studies should be encouraged.

## Pre-publication history

The pre-publication history for this paper can be accessed here:


